# Post-vaccination campaign evaluation of systemic and mucosal immunity of trivalent oral poliovirus vaccine in Karachi, Pakistan (2020–2021): a cross-sectional study

**DOI:** 10.1016/j.lansea.2025.100531

**Published:** 2025-01-21

**Authors:** Ali Faisal Saleem, Visalakshi Jeyaseelan, Zaubina Kazi, Mahjabeen Zehra, Muhammad Masroor Alam, Grace Macklin, Rocio Lopez Cavestany, Sajid Muhammad, Najeeb Rehman, Ondrej Mach

**Affiliations:** a PO Box 3500, Stadium Road, Department of Paediatrics and Child Health, Aga Khan University, 74000, Karachi, Pakistan; b Polio Eradication Department, World Health Organization Headquarters, Avenue Appia 20, 1211, Geneva, Switzerland; c National Institute of Health, Islamabad, Pakistan

**Keywords:** Wild poliovirus, Circulating vaccine-derived polioviruses, Mucosal immunity

## Abstract

**Background:**

The transmission of wild poliovirus (WPV1) and circulating vaccine-derived poliovirus (cVDPV) continues to be a major Public Health Emergency of International Concern. Currently, only Afghanistan and Pakistan remain polio-endemic for WPV1. In response to the co-circulation of VDPV2 with WPV1, the Technical Advisory Group of WHO had recommended two nationwide campaigns of trivalent oral poliovirus vaccine (tOPV) for children aged <5 years in Pakistan in 2020. We assessed the humoral and mucosal immune responses in children who received two doses of tOPV (during vaccination campaigns) in Karachi, Pakistan.

**Methods:**

A cross-sectional survey was conducted in four peri-urban sites (Cattle Colony, Ibrahim Hyderi, Ali Akber Shah, Rehri Goth) Karachi, Pakistan. Venous blood samples from children aged between 1 month and 5 years were obtained. Children who were acutely ill, requiring hospitalisation, with primary immunodeficiency, or with a chronic medical illness, were excluded from the study. Stool and serum testing was performed at the National Institute of Health, Pakistan. Sera samples were analysed using microneutralization assays to quantify polio antibodies for all three serotypes: type 1, 2, and 3. The stool samples collected at baseline, 7, 14, and 28 days (after each tOPV dose) were tested for the presence of poliovirus.

**Findings:**

Of 285 eligible children, 225 had received both tOPV doses and provided three analysable blood samples, and 193 children provided seven viable stool samples. The seroconversion rate for type 2 was 72% (44/61, 95% CI: 59.8–81.8) after the first tOPV dose; cumulative seroconversion after two doses was 93.4% (95% CI: 84.3–97.4). Seven days after the first and second tOPV campaigns, 32.7% and 18.6% excreted Sabin (vaccine) poliovirus type 2, respectively.

**Interpretation:**

The study demonstrated enhanced mucosal immunity as well as a high type 2 seroconversion rate and antibody seroprevalence after two tOPV campaigns.

**Funding:**

10.13039/100004423WHO, Geneva, Switzerland.

**Research in context**

**Evidence before this study**

Previous studies were searched in Google Scholar and PubMed using the keywords “Poliovirus Outbreak response”; “tOPV”; “cVDPV2”; “seroprevalence”; “viral shedding”. Current evidence shows that the transmission of wild poliovirus type 1 (WPV1) and circulating vaccine-derived polioviruses (cVDPV) continues and forms a Public Health Emergency of International Concern. In 2023, there were 11 paralytic cases caused by WPV1 and 370 by cVDPV globally. Afghanistan and Pakistan are the two remaining endemic countries for WPV1 transmission. The WHO Standard Operating Procedures for Response to Polio Outbreaks recommend oral poliovirus vaccine (OPV) to be used due to its ability to induce mucosal immunity in the individual and cease transmission. However, in rare circumstances, the attenuated Sabin virus in OPV can regain neurovirulence and cause outbreaks through person-to-person transmission predominantly in areas with low routine vaccination coverage and hygiene. In 2016, there was a globally synchronized switch of routine use of trivalent OPV (tOPV) to bivalent OPV (bOPV, containing types 1 and 3) to mitigate the risk of cVDPV2 and vaccine-associated paralytic polio from Sabin strains in the vaccine. However, global supply shortages led to several naïve birth cohorts which in turn resulted in waning of type 2 immunity, especially mucosal immunity, and an increased emergence of cVDPV2. In 2020–2021 a large cVDPV2 outbreak was detected in Pakistan resulting in 142 paralytic cases, mainly in Khyber Pakhtunkhwa and Sindh provinces. Due to co-circulation with WPV1, the National Technical Advisory Group recommended responding with tOPV in two tOPV nationwide campaigns targeting children aged <5 years.

**Added value of this study**

The study found out that seroconversion rate for type 2 was 72% (44/61, 95% CI: 59.8–81.8) after the first tOPV dose; cumulative seroconversion after two doses was 93.4% (95% CI: 84.3–97.4) leading to our recommendation of implementing two vaccination campaigns following outbreak detection in Pakistan.

**Implications of all the available evidence**

These data provide useful insights for guiding programmatic efforts in the polio eradication program, specifically in the planning of supplementary immunisation activities. The findings strongly support the recommendation that at least two vaccination campaigns should be implemented in Pakistan as an outbreak response in areas of persistent transmission of wild poliovirus and/or in areas with low immunisation coverage.

## Introduction

After the Global Poliovirus Eradication Initiative (GPEI) was established in 1988, there has been a 99% reduction in poliomyelitis cases worldwide.^1^ The cases of wild poliovirus (WPV) type 2 and 3 were last detected in 1999 and 2012, respectively, and both of these WPV serotypes have been certified as eradicated.^2^^,^^3^ However, the transmission of WPV1 and circulating vaccine-derived polioviruses (cVDPV) continues and forms a Public Health Emergency of International Concern.^4^ In 2023, there were 11 paralytic cases caused by WPV1 and 370 by cVDPV globally.^5^^,^^6^ Afghanistan and Pakistan are the two remaining endemic countries for WPV1 transmission.

The WHO standard operating procedures for response to polio outbreaks recommend oral poliovirus vaccine (OPV) be used due to its ability to induce mucosal immunity in the individual and cease transmission.^7^ However, in rare circumstances, the attenuated Sabin virus strain in OPV can regain neurovirulence and cause outbreaks through person-to-person transmission predominantly in areas with low routine vaccination coverage and hygiene—when this happens, the virus starts to circulate within the community and is referred to as cVDPV.^8^ In 2016, there was a globally synchronised switch of routine use of trivalent OPV (tOPV) to bivalent OPV (bOPV, containing types 1 and 3) to mitigate the risk of cVDPV2 and vaccine-associated paralytic polio from Sabin strains in the vaccine.^9^ Inactivated poliovirus vaccine (IPV) was introduced into routine immunisation schedules to bridge the type 2 immunity gap; however, global supply shortages were leading to several naïve birth cohorts.^10^ This led to the waning of type 2 immunity, especially mucosal immunity, and an increased emergence of cVDPV2.

Since the switch, no live type 2 poliovirus vaccine has been used in routine immunisation anywhere in the world, although monovalent type 2 OPV (mOPV2) and tOPV have been used in supplementary immunisation activities for outbreak response. In 2020–2021 during this study period, a large cVDPV2 outbreak was detected in Pakistan resulting in 142 paralytic cases, mainly in Khyber Pakhtunkhwa and Sindh provinces, with the last case detected in April 2021.^11^ From January to November 2020, there were ten vaccination campaigns with mOPV2 throughout Pakistan; Sindh province experienced two rounds (data from internal WHO Polio Information Services). Due to co-circulation with WPV1, the National Technical Advisory Group recommended responding with tOPV in two tOPV nationwide campaigns targeting children aged <5 years. The tOPV campaigns took place in November 2020 and January 2021.

We conducted a cross-sectional survey among children aged between 1 month and 5 years, with a primary focus on assessing seroconversion for poliovirus type 2 after two outbreak response campaign rounds of tOPV in peri-urban Karachi, Pakistan. Viral shedding was also assessed to provide an indicator of mucosal immunity.

## Methods

This was a facility-based cross-sectional survey conducted in four low-income coastal urban settings of Karachi, Pakistan from October 2020 to February 2021. The areas in Bin Qasim & and Landhi town included Ibrahim Hydri, Ali Akber Shah Goth, Rehri Goth, and Cattle Colony. The Department of Pediatrics of the Aga Khan University (AKU) developed a demographic and surveillance system and runs its primary care and immunisation services. The study protocol was reviewed and approved by the AKU and WHO Ethical Review Committees as well as Pakistan's National Bioethics Committee. The study approval was also sought by the National Emergency Operation Center for Polio Eradication (NEOC, Pakistan). A scientific review was also conducted and approved by the Polio Research Committee at WHO.

### Study population

Children, aged between 1 month and 5 years, residing in the study locality during the vaccination campaign period were included in the study with informed consent of parent or legal guardian. Children who were acutely ill, requiring hospitalisation, with primary immunodeficiency, or with a chronic medical illness, were excluded from the study. A target sample size of 250 children was calculated based on 40% type 2 seroprevalence, a 20% attrition rate, a design effect of 1.5, and a 95% confidence margin. Children were identified using simple random sampling from AKU's Demographic Health Surveillance Survey (DSS) database and invited by the community health workers to enroll in the study at the nearest AKU health centre. The DSS is based on five low-income settlements in Karachi near the coastal belt. The DSS collects routine data on household demographics, including age, gender, household structure, vaccination history, pregnant household, and health status. These data are collected and updated bi-annually and cover all age groups. However, there are regular health demographics updates, particularly for newborns and mortality. Sociodemographic information (i.e., parent's age, education status), child characteristics (i.e., age, categorized as ≤2 years and >2 years old, gender, birth weight), sanitation measures (i.e., drinking water source, type of toilet), and complete vaccination history was obtained during enrolment. Malnutrition indicators including wasting (weight-for-height) and stunting (height-for-age) were calculated.

### Biological sample collection, processing, and laboratory analysis

Baseline blood and stool samples were collected before the first tOPV campaign. After each of the two tOPV campaigns, blood was collected 28 ± 3 days, and stool was collected 7 ± 1 days, 14 ± 1 days, and 28 ± 1 days after vaccine administration. A total of 3 blood samples and 7 stool samples were collected from each enrolled child.

Blood samples (2 mL) were collected by a trained phlebotomist via peripheral venipuncture. Samples were then transported to AKU for processing and serum preparation. The samples were stored at −80 °C in the Infectious Diseases Research Laboratory, until shipment to National Institute of Health (NIH), Islamabad, where the blood samples were analysed using standard microneutralization assays against polio antibodies for all three serotypes.^12^ The maximum dilution tested was 1:1024 (and the highest detectable titre reported was 1 ≥ 1448); the minimum (nondetectable) titre reported was <1:8. Stool samples were tested for the presence of poliovirus using the standard viral culture technique and intertypic differentiation at the NIH, Islamabad.^13^

### Outcome definitions

The primary outcomes were seroconversion for type 2 poliovirus antibodies. Seropositivity was defined as the presence of neutralising antibody titres in dilution ≥1:8^12^; antibody titres are presented in the log_2_ scale. Seroconversion was defined as individuals changing from seronegative to seropositive after a tOPV campaign. Boosting was defined as individuals seropositive at baseline who experienced a four-fold increase in titres.^14^ The presence of mucosal immunity was defined as the absence of viral shedding in stool 7 days after the OPV challenge which mimics live viral infection.^15^^,^^16^ Children aged <6 months (19, 6.7%) were excluded from the analysis of seroconversion, boosting, and median titres due to interference with maternal antibodies.

### Statistical analysis

The data was analysed using STATA v.15 and R. Missing data were addressed in real-time due to the use of Electronic Data Capture (EDC). If any data remained missing, a special code was assigned to ensure they were included in the analysis or dataset. These missing values are incorporated in descriptive statistics for categorical data, but they are excluded from continuous data analyses. Descriptive analysis for sociodemographic variables was performed by calculating frequency with percentages for categorical data and mean with standard deviation or median with interquartile ranges for continuous scale data. Age-specific proportions with 95% confidence intervals were calculated for seroprevalence, seroconversion, and viral shedding. Antibody titres are presented with median and 95% bootstrap confidence intervals. The distribution of titres was compared using the log-rank test. Sociodemographic characteristics were associated with seroprevalence using Fisher's exact test. Generalized linear models with logit transformation were applied to assess the factors associated with type 2 seroconversion and immune shedders.

### Role of the funding source

The funders had no role in conduct of the study, interpretation, writing the manuscript or decision to submit. No authors were paid to write this article by any company, organisation or agency.

## Results

**Fig. 1:**
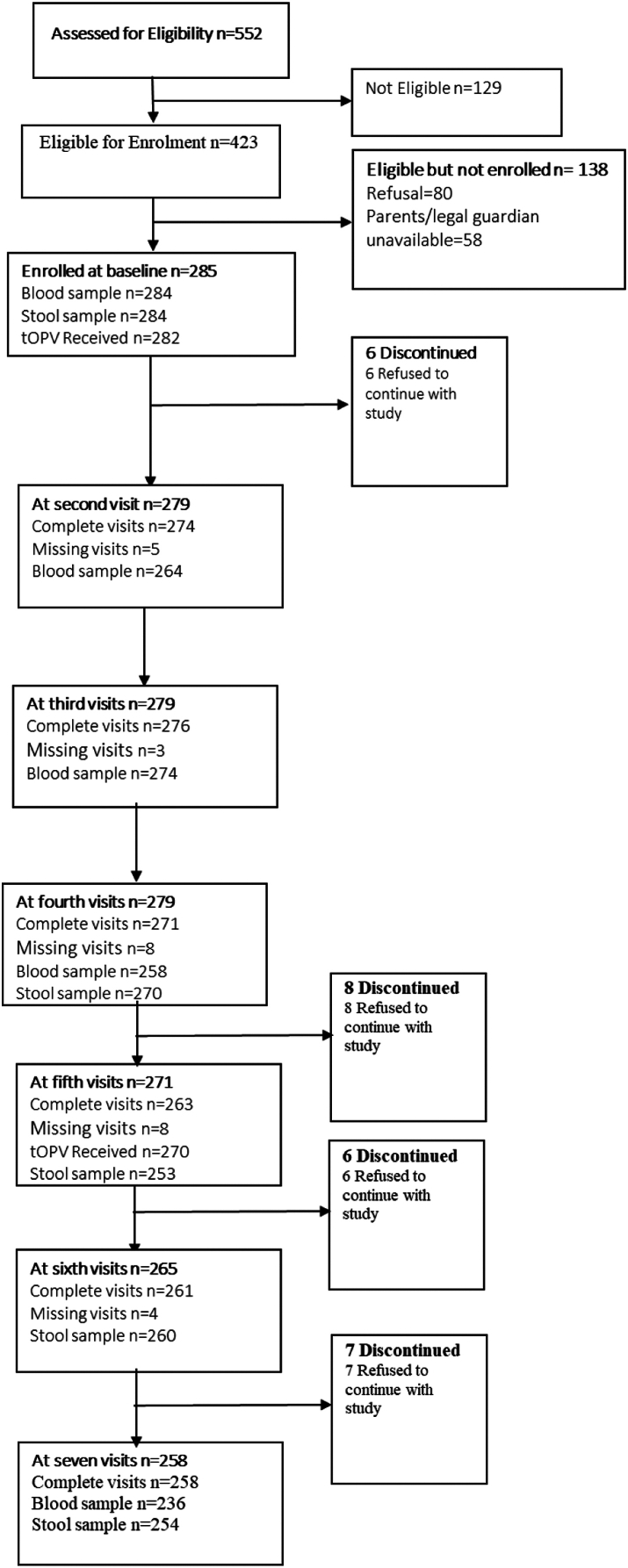
**Study participants enrolment**.

A total of 552 children were screened, of which 285 were eligible and enrolled in the study (Fig. 1). There were 80 refusals, 58 unavailable parent or legal guardians, and 129 non-eligible children (outside of age range [1 month–5 years], not remaining in the study area for the duration of research procedures, child found acutely ill requiring hospitalisation or presenting with primary immunodeficiency or another chronic medical illness). Of the 285 enrolled children, 282 received the first round of tOPV and 270 received the second round. Children who received both tOPV doses and had three viable blood samples collected were 225, and we present the data obtained from these children. In addition, children who received both tOPV doses and provided seven viable stool samples were 193.

### Sociodemographic variables

Baseline sociodemographic variables are summarised in Table 1. Of 225 children, 49% (110/225) were male and the average age at enrolment was 32.2 months (1–57 months). Vaccination history was recorded either through an available vaccination card (60/225, 27%) or via parental recall (156/225, 70%); 8% (19/225) had an unknown vaccination history. One dose of IPV was received by 76% (156/206) of participants. Four doses of bOPV in routine immunisation were received by 82% (184/225) children; 7% (16/225) received 0 doses of bOPV. The collected values for weight, height, and age were used to calculate malnutrition indicators: 11% (24/218) experienced wasting, 44% (94/215) stunting and 35% (78/222) were underweight.

**Table 1:** Baseline sociodemographic characteristics of children with three analysable blood samples (N = 225).

Baseline characteristics	Number (%)
Total	225 (100%)
**Gender**	
Male	110/225 (48.9%)
**Age in months at enrolment**	
Median (range)	32.2 (1–57)
**Vaccination history**	
Vaccination card	60/225 (26.7%)
Recall	156/225 (69.3%)
Unknown	19/225 (8.4%)
**IPV received (one dose)**	156/206 (75.7%)
**Number bOPV (RI)**	
Full schedule (Birth + 3 doses)	184/225 (82.0%)
0 doses	16/225 (7%)
**Nutritional indicators**	
Wasting (weight-for-height)	24/220 (10.9%)
Stunting (height-for-age)	99/222 (44.6%)
Underweight	78/222 (35.1%)
**Mother's education level**	
No formal education	123/225 (54.7%)
Religious education	5/225 (2.2%)
Completed primary	35/225 (15.6%)
Completed secondary	45/225 (20.0%)
**Father's education level**	
No formal education	133 (59.1%)
Religious education	8 (3.6%)
Completed primary	22 (9.8%)
Completed secondary	45 (20.0%)

bOPV, bivalent oral poliovirus vaccine; IPV, inactivated poliovirus vaccine; RI, routine immunisation.

### Serological analysis

**Fig. 2:**
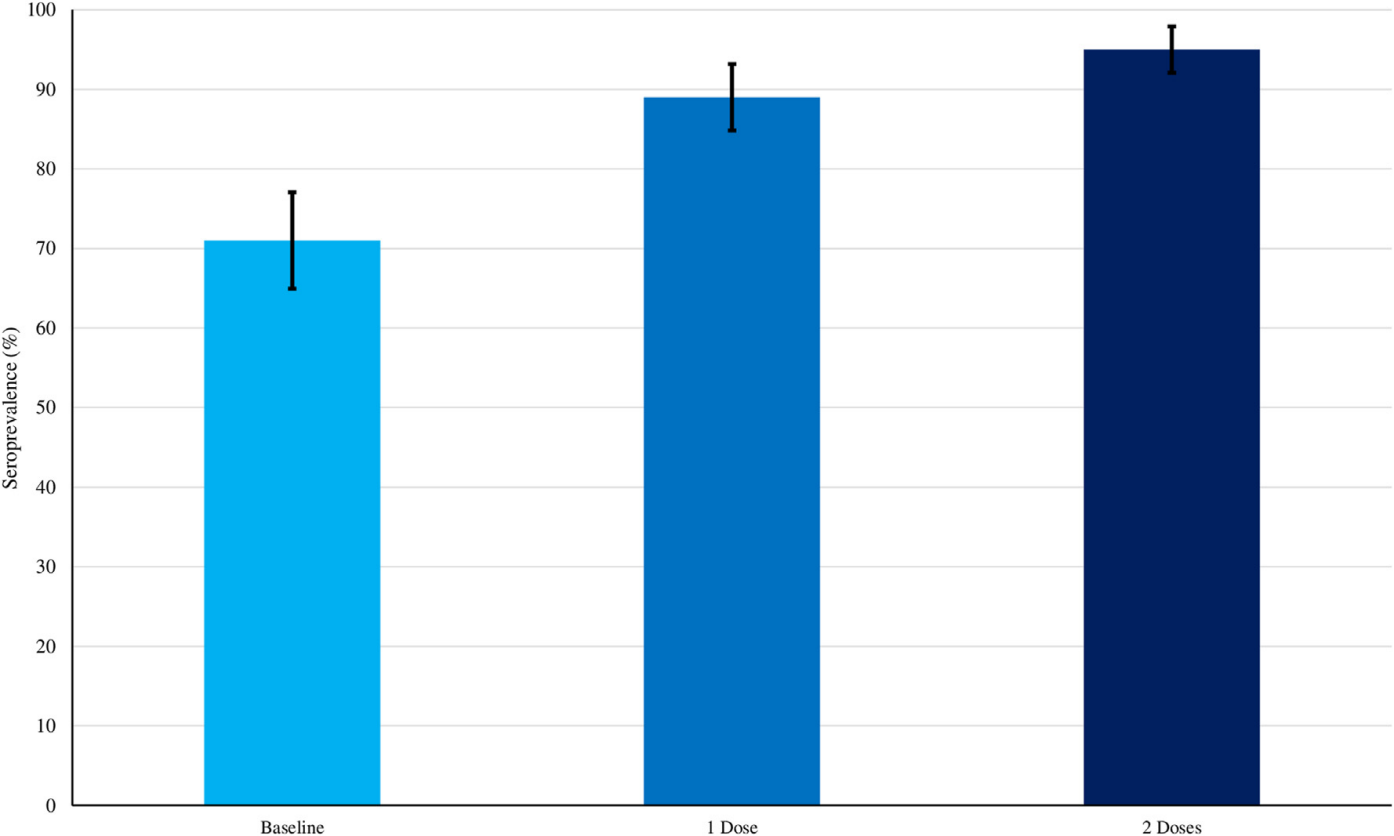
**Overall type 2 seroprevalence at baseline (enrolment), 28 days after the first trivalent oral poliovirus vaccine (tOPV) dose and 28 days after second tOPV dose, N = 225**.

**Fig. 3:**
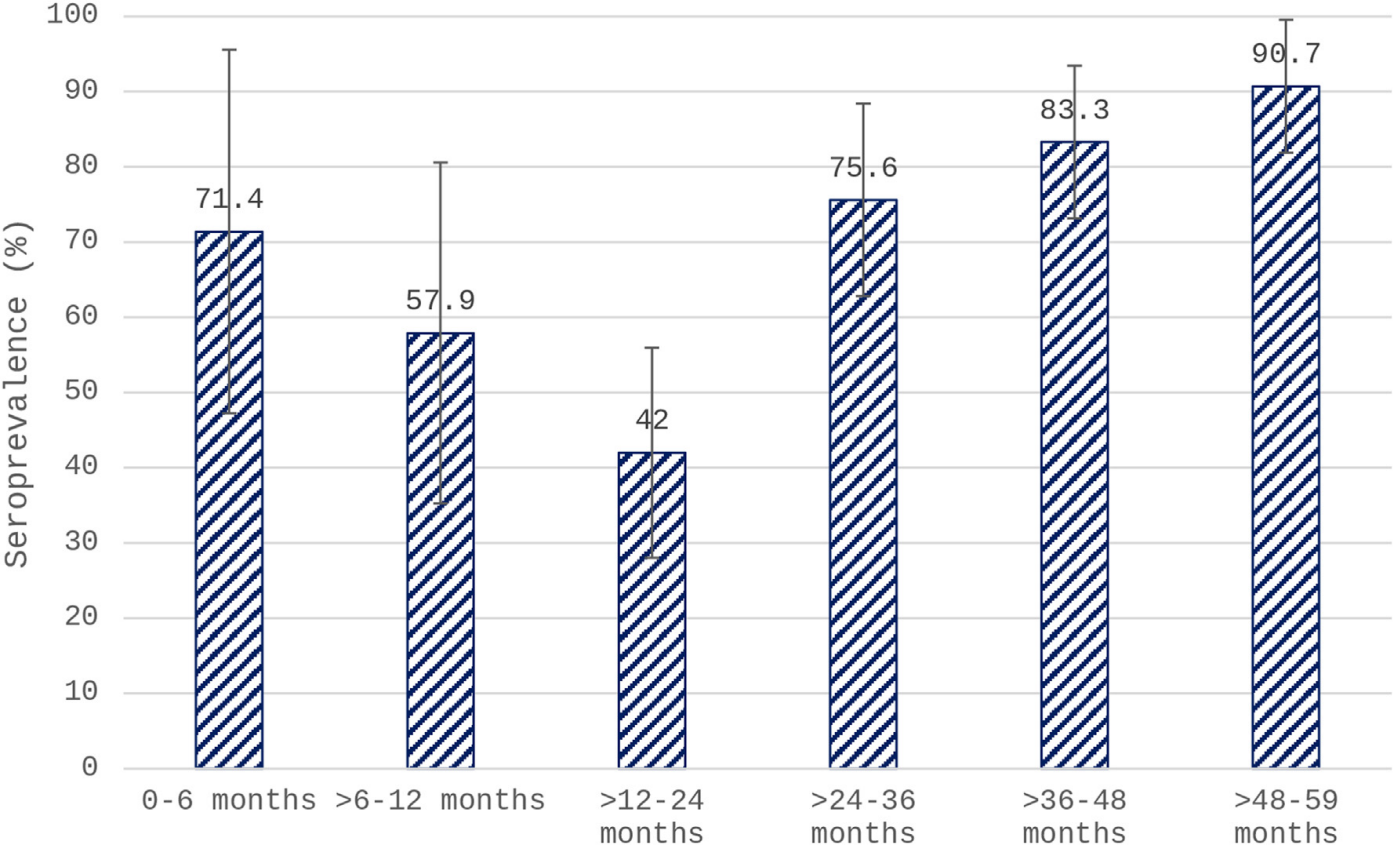
**Baseline type 2 seroprevalence by age group (N = 225). 0–6 months (mo), n = 14; >6–12 mo, n = 19; >12–24 mo, n = 50; >24–36 mo, n = 45; 36–48 mo, n = 54; >48–59 mo, n = 43**.

Type 2 seroprevalence was 71.1% (95% CI: 64.7–76.9) at baseline, 89% (95% CI: 84–92.7) after one tOPV dose, and 95% (95% CI: 91.4–97.5) after two tOPV doses (Fig. 2). Baseline seroprevalence for type 2 stratified by age is presented in Fig. 3. The types 1 and 3 baseline seroprevalences were 95.1% (95% CI: 91.5–97.2) and 81.8% (95% CI: 76.2–86.3) respectively. The type 1 and type 3 seroprevalences after the first and second tOPV campaigns were 96.9% (95% CI: 93.7–98.5), 99.1% (95% CI: 96.8–99.8); 90.2% (95% CI: 85.6–93.5), 92.9% (95% CI: 88.8–95.6) respectively.

Type 2 seroconversion, boosting, and titre analysis included children above 6 months of age (n = 210); seroconversion and boosting response are summarised in Table 2. There were 61 participants seronegative at baseline, of which 72% (44/61, 95% CI: 59.8–81.8) seroconverted after the first tOPV dose. The cumulative two-dose seroconversion rate was 93.4% (57/61, 95% CI: 84.3–97.4).

**Table 2:** Type 2 seroconversion and boosting.

	Time period	% (n/N)	95% CI
Seroconversion	1st dose seroconversion (Baseline to Visit 1)	72.1% (44/61)	59.8–81.8
	Two-dose seroconversion	93.4% (57/61)	84.3–97.4
Boosting	1st dose boosting (Baseline to Visit 1)	51.4% (57/111)	42.2–60.4
	Two-dose boosting	44.1% (49/111)	34.7–53.9

**Fig. 4:**
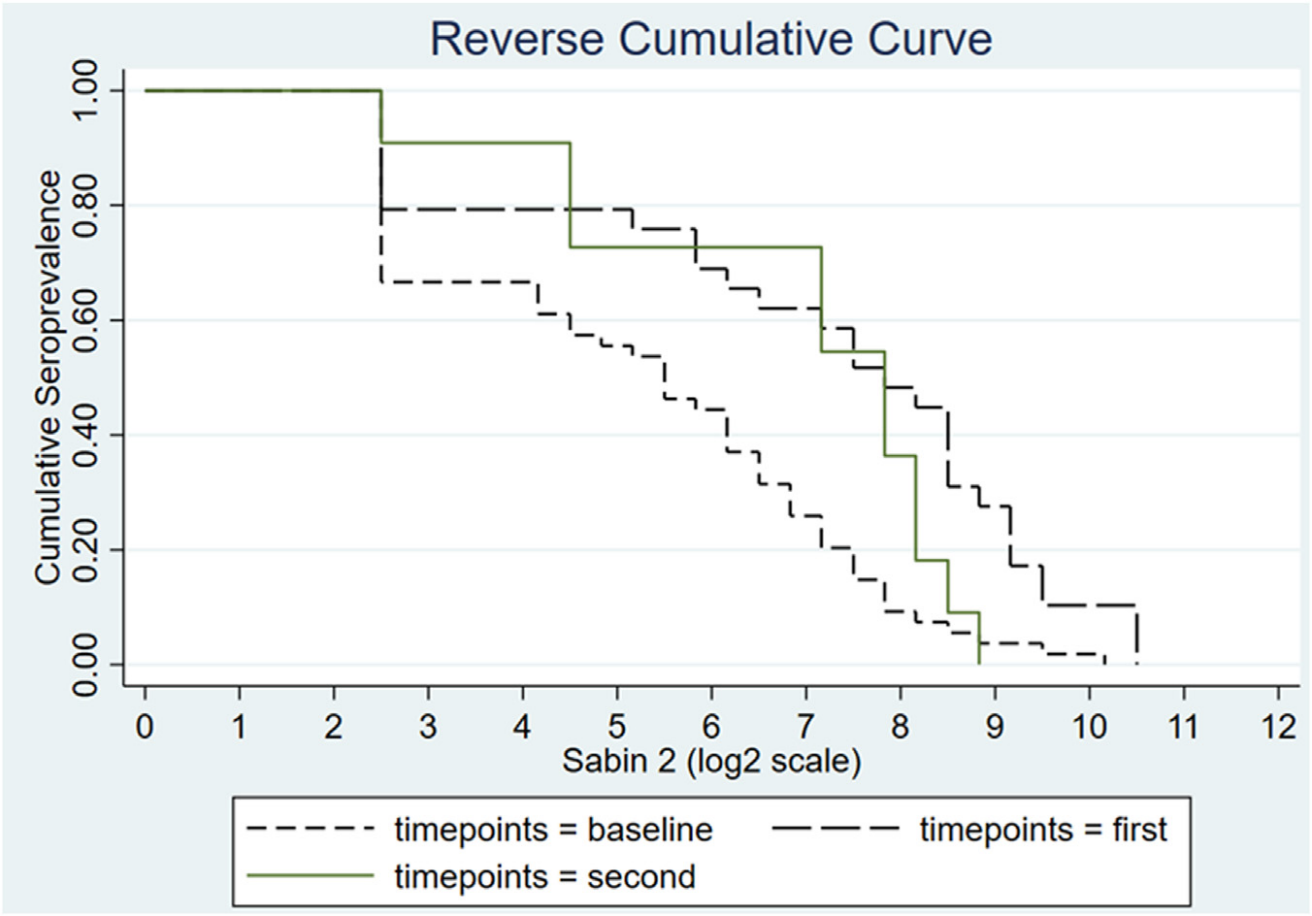
**Reverse cumulative curve for type 2 antibody titres at baseline (visit 1) and after the first and second tOPV dose (visit 2 and 3, respectively). Median with 95% CI reciprocal titres is expressed in log**_**2**_**scale. Children aged <6 months were excluded due to interference with maternal antibodies**.

Median type 2 antibody titres were 6.2 (95% CI: 5.5–6.5) at baseline, 8.8 (95% CI: 8.2–9.2) after one tOPV dose, and 7.8 (95% CI: 7.5–8.2) after the second tOPV dose. The median titre after the second tOPV dose was significantly lower than the median titre after the first tOPV dose (p = 0.016). The reverse cumulative curve for type 2 antibody titres is depicted in Fig. 4.

### Viral stool shedding analysis

**Fig. 5:**
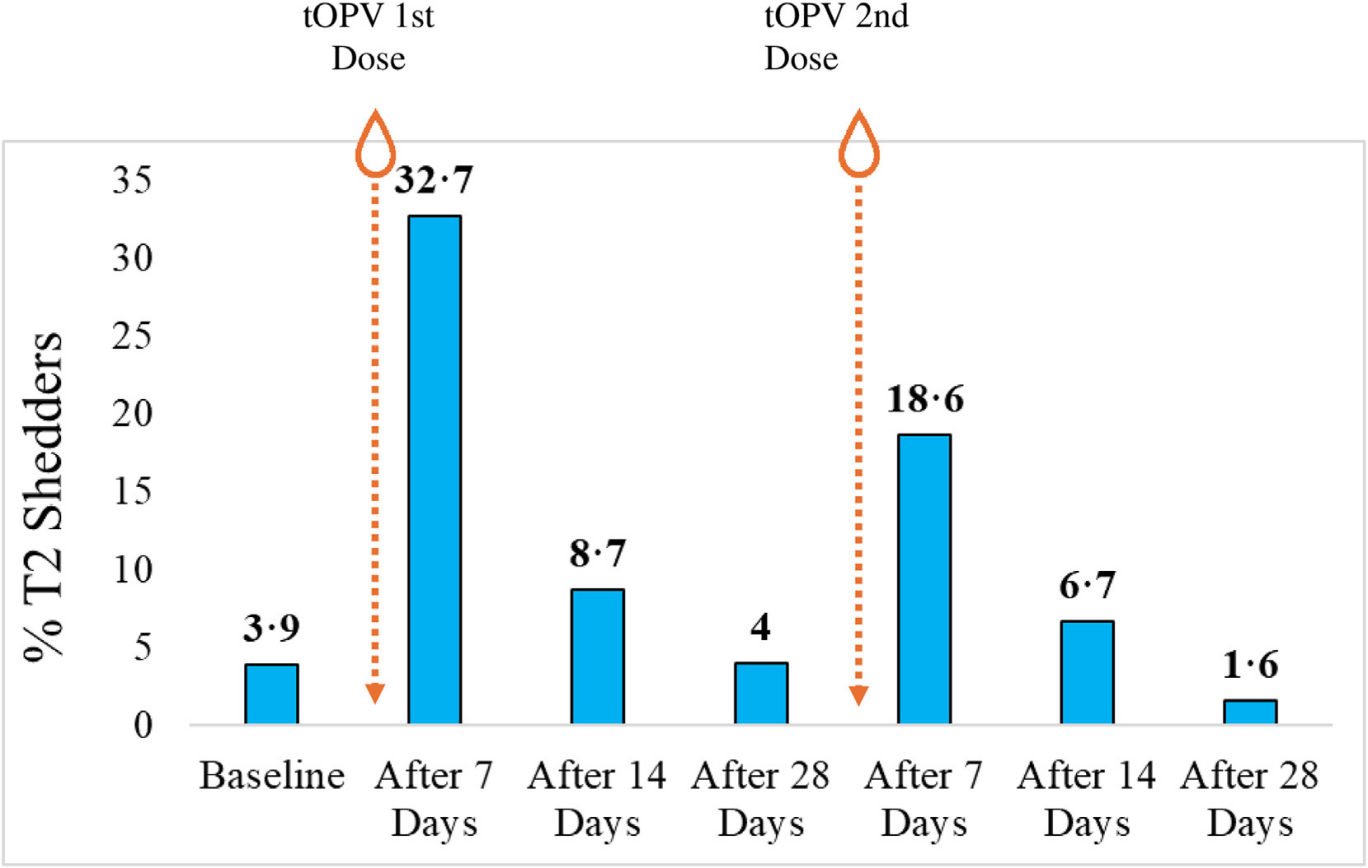
**Shedding of Sabin poliovirus type 2 in relation to the first and second trivalent oral poliovirus vaccine (tOPV) campaign**.

Children included in the stool shedding analysis had analysable stool samples collected at all time points and received both tOPV doses (n = 193). The prevalence of viral shedding is presented in Table 3 and Fig. 5. At baseline, the excretion rates of Sabin-like poliovirus type 1 (SL1) and SL3 were 1.2% (3/259) each, whereas the excretion rate of SL2 was found to be 3.9% (10/259) at baseline. No children were excreting WPV1. 32.7% (71/217) and 18.6% (34/183) excreted SL2 7 days post first and second tOPV campaigns respectively. 1.8% (4/217) children excreted SL1 7 days after the first tOPV dose and there were no viruses shed 28 days post first and second doses of tOPV. Similarly, there were no SL3 viruses shed 28 days after the first and second doses of tOPV. Among the seropositive individuals, 34% and 20% shed SL2 following the 1st and 2nd tOPV campaigns, respectively, while 36% of seronegative individuals shed SL2 after both campaigns. Among those seropositive at baseline, 38 out of 161 (23.6%) shed SL2 after the first tOPV dose, compared to 32 out of 161 (19.9%) of seronegative individuals who did not shed SL2, with no statistically significant difference. After the second tOPV dose, 17.8% of seropositive children shed SL2, while only 5.9% of seronegative children did not (Table S2 and Table S3).

**Table 3:** Viral shedding patterns after the two trivalent oral poliovirus vaccine (tOPV) campaigns.

	Baseline (N = 259)	7 days post 1st tOPV dose (N = 217)	14 days post 1st tOPV dose (N = 207)	28 days post 1st tOPV dose (N = 174)	7 days post 2nd tOPV dose (N = 183)	14 days post 2nd tOPV dose (N = 209)	28 days post 2nd tOPV dose (N = 192)
	n (%)	n (%)	n (%)	n (%)	n (%)	n (%)	n (%)
SL1	3 (1.2)	4 (1.8)	1 (0.5)	0	6 (3.3)	2 (1.0)	0
SL2	10 (3.9)	71 (32.7)	18 (8.7)	7 (4.0)	34 (18.6)	14 (6.7)	3 (1.6)
SL3	3 (1.2)	9 (4.1)	4 (1.9)	0	9 (4.9)	8 (3.8)	0
NPEV	39 (15.1)	23 (10.6)	20/206 (9.7)	24/173 (13.9)	23 (12.6)	45/208 (21.6)	27/192 (14.1)

SL, Sabin-like; SL1/2/3, Sabin-like poliovirus type 1/2/3; tOPV, trivalent oral poliovirus vaccine. NPEV, Non-polio enterovirus.

### Risk factor analysis

The bivariate analysis for seroconversion and immune shedders is presented in the supplementary information (Table S1). There was no statistically significant association between sociodemographic or nutritional indices and seroconversion.

## Discussion

After two national tOPV campaigns, we observed high type 2 seroprevalence (95.1%). These observations align with previous studies conducted both before and after the tOPV-bOPV switch. Median titres of type 2 antibodies and titre distribution were high. The seroprevalence against types 1 and 3 was high with high median titres at baseline (95.1% and 81.8%) and after one (96.9% and 99.1%) and two (90.2% and 92.9%) tOPV rounds due to bOPV and IPV from routine immunisation (RI).

The study revealed a high baseline type 2 seroprevalence (71.1%) which likely results from a combination of IPV from RI and exposure to cVDPV2. IPV coverage has increased since the tOPV-bOPV switch, reaching 90% in 2019. Nevertheless, there was no observed association between the history of IPV vaccination, mostly relying on parental recall and the prevalence of type 2 seropositivity. The highest seroprevalence of type 2 was observed in the eldest age group (>48 months), which is likely due to IPV from RI and a greater number of vaccination campaigns received. The lowest seroprevalence was observed in children aged 12–24 months, which may be attributed to the complete disappearance of maternal antibodies.

The stool examination did not yield any incidental findings of WPV1. This demonstrates no underlying WPV1 transmission in the population enrolled in this study. The incidence of SL1 excretion was very low in this study as compared to a previous study^17^ after the bOPV challenge (1.8% vs 13.7%), suggesting a substantial improvement in type 1 mucosal immunity within the studied population. But at the baseline, there was increased excretion of SL2 by the study participants, clearly indicating previous exposure to type 2 poliovirus.

There was no statistically significant difference in viral shedding between children who tested seropositive and seronegative. Younger children (aged ≤2 years) who had recently received IPV or tOPV may have stronger mucosal immunity, while older children (aged >2 years) may experience waning immunity. The mixed cohort may have diluted the observed relationship between seropositivity and viral shedding, as the reduced immunity in older children may be less effective in preventing intestinal viral replication.

No sociodemographic risk factors were found to be associated with type 2 seroconversion, most likely because the sample size was insufficient to demonstrate associations, and similar results were reported from surveys conducted in Cameroon, Liberia, and Tajikistan.^18^, ^19^, ^20^

The current study has several strengths. The research sample was taken from the urban regions of Karachi where most of the population belongs to a low socioeconomic class and has poor water, sanitation, and hygiene (WASH) practices. Despite poor card retention, the study population has high RI coverage as confirmed by their serum and mucosal immunity. We also noticed that some people refused to participate in the study due to their apprehensions regarding polio-related research. The results of the study support the decisions of national advisory technical group of experts on immunizations (NITAG), NEOC, and WHO for controlling cVDPV2 in an outbreak situation. Use of a moderate sample size though may be considered a limitation of the study.

Compelling evidence presented in this research supports the recommendation to implement two campaigns following an outbreak detection. This is especially important in areas with inadequate vaccine coverage or persistent wild poliovirus transmission, as indicated by a significant increase in seroconversion or seroprevalence rates. The Strategic Advisory Group of Experts (SAGE) recommends conducting an IPV (full or fractional dose) campaign in conjunction with an OPV campaign in Pakistan. This is because IPV enhances individual-level protection and decreases transmission by improving mucosal immunity.^21^

These data provide useful insights for guiding programmatic efforts in the polio eradication program, specifically in the planning of SIAs. The findings strongly support the recommendation that at least two campaigns should be implemented as an outbreak response in areas of persistent transmission of wild poliovirus and/or in areas with low immunisation coverage.

## Contributors

Conceptualisation: AFS, OM. Data Curation: MZ, NR, SM. Formal analysis: VJ, SM, NR, RLC, GM. Funding Acquisition: AFS, ZK. Methodology: AFS, AK, MZ. Project administration: MZ, ZK. Resources: MMA. Supervision: AFS, OM. Visualisation: VJ, GM, RLC. Writing-Original Draft and Writing-Review and editing: AFS, MJ, VJ.

## Data sharing statement

Due to ethical and privacy concerns, raw data containing personal identifiers are not publicly available. However, anonymized data will be shared upon reasonable request and subject to approval by the relevant ethics committee. For data access, please contact the corresponding author.

## Editor note

The Lancet Group takes a neutral position with respect to territorial claims in published maps and institutional affiliations.

## Declaration of interests

We declare no competing interests.
