# Supplementary material for: Post-vaccination campaign evaluation of systemic and mucosal immunity of trivalent oral poliovirus vaccine in Karachi, Pakistan (2020–2021): a cross-sectional study
Source: Lancet Reg Health Southeast Asia. 2025 Jan 21;33:100531. doi: 10.1016/j.lansea.2025.100531 (PMC11788853; doi:10.1016/j.lansea.2025.100531)

**Supplementary information**

*tOPV (trivalent Oral Polio vaccine); **IPV (Injectable Poliovirus)

**Table S1: Bivariate analysis of socio-demographic characteristics with type 2 seroconversion, and immune shedders after first and second tOPV doses**

| **Variables** | | **Type 2 Seroconversion**  **n/N (%)** | **P value** | **Immune Shedders** | | | |
| --- | --- | --- | --- | --- | --- | --- | --- |
|  |  |  |  | **After 1st**  **tOPV dose** | **P value** | **After 2nd**  **tOPV dose** | **P value** |
|  |  |  |  | **n/N (%)** |  | **n/N (%)** |  |
| **Age** | ≤ 2 years | 36/37 (97·3) | 0·29 | 21/51 (41·2) | 0·29 | 13/55 (23·6) | 0·68 |
|  | >2 years | 21/24 (87·5) |  | 35/110 (31·8) |  | 19/97 (19·6) |  |
| **Gender** | Male | 25/27 (92·6) | 1·00 | 28/73 (38·4) | 0·41 | 14/76 (18·4) | 0·55 |
|  | Female | 32/34 (94·1) |  | 28/88 (31·8) |  | 18/76 (23·7) |  |
| **Wasting** | Normal | 51/55 (92·7) | 1·00 | 51/146 (34·9) | 0·75 | 29/137 (21·2) | 1·00 |
|  | Wasted | 5/5 (100) |  | 3/11 (27·3) |  | 3/14 (21·4) |  |
| **Stunted** | Normal | 24/25 (96·0) | 0·64 | 27/83 (32·5) | 0·74 | 16/77 (20·8) | 1·00 |
|  | Wasted | 33/36 (91·7) |  | 27/75 (36·0) |  | 16/74 (21·6) |  |
| **OPV doses** | 0 doses^*^ | NA | NA | 8/11 (61·5) | 0·064 | 0/11 (0) | 0·07 |
|  | Full schedule^**^ (birth+3 doses) |  |  | 45/136 (33·1) | 0·361 | 27/128 (21·1) | 1·00 |
| **IPV received** | Yes | 35/38 (92·1) | 1·00 | 38/114 (33·3) | 0·84 | 24/107 (22·4) | 0·81 |
|  | No | 14/15 (93·3) |  | 12/33 (36·4) |  | 6/31 (19·4) |  |

^*^Reference is ≥1 dose; ** Reference is <3 doses

**Figure S1.** Overall seroprevalence of all three types at baseline (enrolment), 28 days after the first tOPV dose and 28 days after second tOPV dose, N=225. tOPV, trivalent oral poliovirus vaccine.

|  | SL2 found in stool 7 days after first tOPV | |  |  |
| --- | --- | --- | --- | --- |
|  | **Present** | **Absent** |  |  |
| **Prior Type 2 seroprevalence** | **n** | **n** | **Total** |  |
| Seropositive | 38  23.6% | 73  45.3% | 111 | 34% of seropositive are shedders |
| Seronegative | 18  11.2% | 32  19.9% | 50 | 36% of seronegative are shedders |
| Total | 56 | 105 | **161** | P= 0.805 |
|  | 68% of shedders are seropositive | 70% of non-shedders are seropositive | P= 0.793 |  |

**Table S2**: Association of seroprevalence and shedding after first tOPV campaign

|  | SL2 found in stool 7days after 2^nd^ tOPV | |  |  |
| --- | --- | --- | --- | --- |
|  | **Present** | **Absent** |  |  |
| **Prior Type 2 seroprevalence** | **n** | **n** | **Total** |  |
| Seropositive | 27  17.8% | 111  73.0% | 138 | 20% of seropositive are shedders |
| Seronegative | 5  3.2% | 9  5.9% | 14 | 36% of seronegative are shedders |
| Total | 32 | 120 | **152** | P= 0.165 |
|  | 84% of shedders are seropositive | 92% of non-shedders are seropositive | P= 0.174 |  |

**Table S3**: Association of seroprevalence and shedding after second tOPV campaign

| IPV history | Type 2 | |  |
| --- | --- | --- | --- |
|  | Seroprevalent at baseline | Not seroprevalent at baseline | P value |
| Received IPV | 74% (115/156) | 26.3% (41/156) | 0.469 |
| Not received IPV | 32.0% (16/50) | 68.0% (34/50) |  |

**Table S4**: Association of type 2 seroprevalence and IPV history

| Demographic variables | Children who had 2 tOPV doses and 7 stool samples (included for analysis) | | Children who were not included for analysis | |
| --- | --- | --- | --- | --- |
|  | **n** | **%** | **n** | **%** |
| Age of the child (in months) |  |  |  |  |
| 0-6 | 8 | 4.6 | 11 | 10.0 |
| >6-12 | 15 | 8.6 | 12 | 10.9 |
| >12-24 | 41 | 23.4 | 23 | 20.9 |
| >24-36 | 35 | 20.0 | 22 | 20.0 |
| >36-48 | 46 | 26.3 | 21 | 19.1 |
| >48-59 | 30 | 17.1 | 21 | 19.1 |
| Male children | 83 | 47.4 | 54 | 49.1 |
| Stunted | 84 | 48.3 | 44 | 40.7 |
| Wasted | 15 | 8.7 | 21 | 15.3 |
| Underweight | 59 | 33.9 | 43 | 39.8 |

**Table S5**: Map of Karachi, Pakistan showing the distribution of children included and not included in the analysis by demographic variables and nutritional indices

**Site Map:**


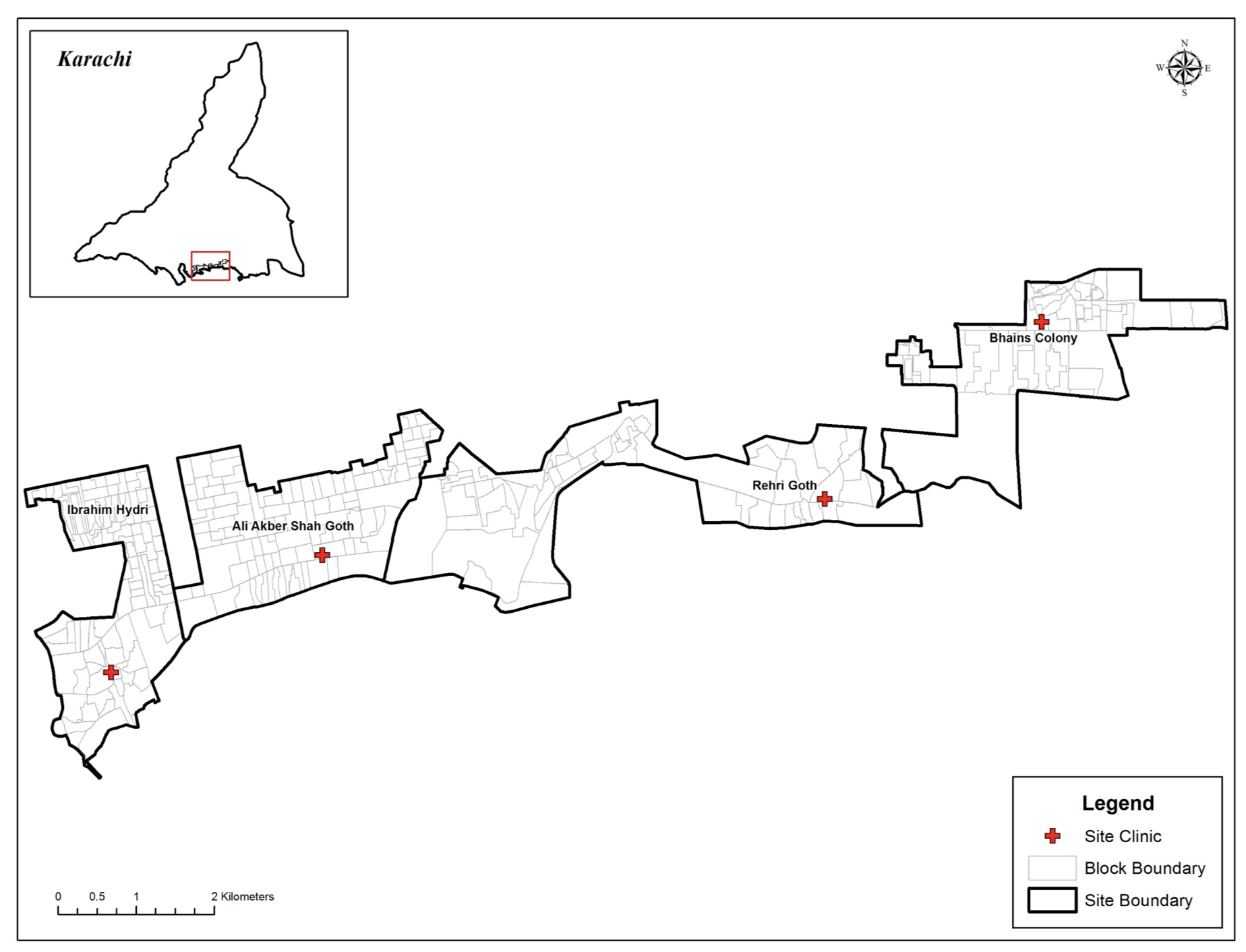

Supplement: Supplementary Tables [file mmc1.docx]
